# Tuberculosis Among Foreign-Born Persons Diagnosed ≥10 Years After Arrival in the United States, 2010–2015

**DOI:** 10.15585/mmwr.mm6611a3

**Published:** 2017-03-24

**Authors:** Clarisse A. Tsang, Adam J. Langer, Thomas R. Navin, Lori R. Armstrong

**Affiliations:** 1 Division of TB Elimination, National Center for HIV/AIDS, Viral Hepatitis, STD, and TB Prevention, CDC.

The majority of tuberculosis (TB) cases in the United States are attributable to reactivation of latent TB infection (LTBI) (*1*). LTBI refers to the condition when a person is infected with *Mycobacterium tuberculosis* without signs and symptoms, or radiographic or bacteriologic evidence of TB disease. CDC and the U.S. Preventive Services Task Force (USPSTF) recommend screening populations at increased risk for LTBI, including persons who have lived in congregate settings at high risk and persons who were born in, or are former residents of countries with TB incidence ≥20 cases per 100,000 population (*2*–*4*). In 2015, foreign-born persons constituted 66.2% of U.S. TB cases (*5*). During the past 30 years, screening of persons from countries with high TB rates has focused on overseas screening for immigrants and refugees, and domestic screening for persons who have newly arrived in the United States (*6*,*7*). However, since 2007, an increasing number and proportion of foreign-born patients receiving a diagnosis of TB first arrived in the United States ≥10 years before the development and diagnosis of TB disease. To better understand how this group of patients differs from persons who developed TB disease and received a diagnosis <10 years after U.S. arrival, CDC analyzed data for all reported TB cases in the United States since 1993 in the National TB Surveillance System (NTSS). After adjusting for age and other characteristics, foreign-born persons who arrived in the United States ≥10 years before diagnosis were more likely to be residents of a long-term care facility or to have immunocompromising conditions other than human immunodeficiency virus (HIV) infection. These findings support using the existing CDC and USPSTF recommendations for TB screening of persons born in countries with high TB rates regardless of time since arrival in the United States (*2*,*3*).

In the NTSS, persons are categorized as foreign-born if they were born outside of the United States, U.S. insular* areas, and the freely associated states^†^ (except persons born abroad to a U.S. citizen parent). The number of years in the United States is defined as the interval from first entry into the United States to the date the TB patient was first reported to a health department. Persons were classified as having arrived in the United States <10 years or ≥10 years before diagnosis. Persons missing month or year of U.S. entry were excluded from the analysis when comparing the two groups. Persons <10 years of age were also excluded from the comparison analysis because they could not have lived in the United States for ≥10 years. Adjusted odds ratios were calculated using a logistic regression model and backward elimination of variables with statistically insignificant effects (p>0.05) in the model to assess the association between receiving a diagnosis of TB disease ≥10 years after U.S. entry compared with <10 years after U.S. entry and a demographic characteristic or TB risk factor. Age at diagnosis was modeled categorically and divided into 10-year groups.

During 1993–2015, the number and proportion of TB cases among foreign-born persons who were missing month or year of U.S. entry declined from 2,689 (36.3%) to 587 (9.2%), and the number and proportion of TB cases among foreign-born persons who arrived in the United States ≥10 years before diagnosis increased from 1,360 (18.4%) in 1993 to 2,922 (46.0%) in 2015 (Figure). During 2010–2015, 38,345 new cases of TB were reported among foreign-born persons, 34,866 (90.9%) of whom had complete U.S. entry date information. During 2010–2015, among all foreign-born persons with TB disease, the median interval from arrival in the United States to developing TB was 9 years, (interquartile range [IQR] = 2–21 years); the median age at arrival was 29 years (IQR = 21–43 years), and the median age at TB diagnosis was 45 years (IQR = 30–62 years). Among foreign-born persons with TB diagnosed after residing ≥10 years in the United States, the median time spent in the United States before developing TB was 21 years (IQR = 14–31 years) compared with 2 years (IQR = 0–5 years) among persons who resided in the United States <10 years. The median age at arrival for both TB patients who had been in the United States ≥10 years and <10 years before diagnosis was 29 years (IQR = 20–42 years, IQR = 22–44 years, respectively). The median age at TB diagnosis was 56 years (IQR = 43–69 years) for persons with TB diagnosed after ≥10 years in the United States, compared with 33 years (IQR = 25–48 years) for persons with TB diagnosed <10 years in the United States. The top three countries of origin for persons with TB diagnosed ≥10 years after U.S. arrival were Mexico (26.8%), the Philippines (14.0%), and Vietnam (9.2%), whereas the top three countries of origin among persons with diagnoses <10 years after U.S. arrival were Mexico (14.3%), India (10.6%), and the Philippines (10.3%). After adjusting for other factors in the multivariable model, ≥10-year residents were significantly more likely to be aged ≥40 years and to report being of Hispanic ethnicity (Table). Similarly, ≥10-year residents were independently associated with residing in a long-term care facility at diagnosis, reporting excess alcohol use during the year preceding diagnosis, and having a history of a non-HIV–related immunocompromising condition, including diabetes mellitus, end-stage renal disease, tumor necrosis factor-alpha antagonist therapy, or having received an organ transplant (Table). However, ≥10-year residents had lower odds of being a resident of a correctional facility at the time of diagnosis (Table).

**FIGURE:**
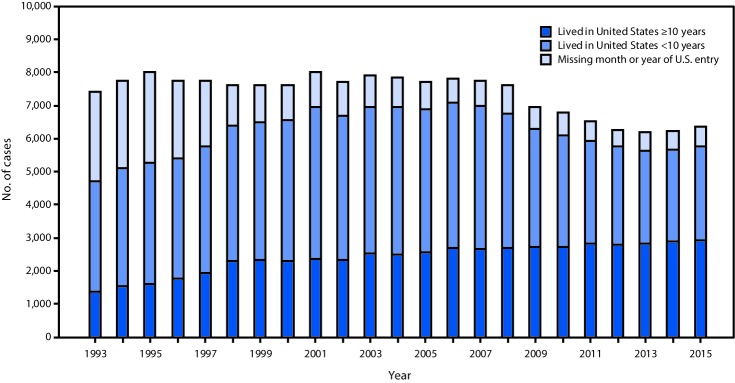
Number of tuberculosis cases diagnosed among foreign-born persons <10 years and ≥10 years after arrival in the United States, 1993–2015

**TABLE:** Characteristics and adjusted odds ratios of foreign-born patients receiving a tuberculosis (TB) diagnosis ≥10 years versus <10 years after arrival in the United States, 2010–2015*

Characteristic	No. (%) TB cases		Adjusted odds ratio (95% CI)^†^
	Diagnosed <10 years after U.S. arrival (n = 17,492)	Diagnosed ≥10 years after U.S. arrival (n = 16,989)	
**Sex**			
Male	9,826 (56.2)	10,390 (61.2)	1.1 (1.0–1.2)
Female	7,663 (43.8)	6,595 (38.8)	Referent
**Race/ethnicity** ^§^			
Black	3,445 (19.7)	1,342 (7.9)	0.5 (0.4–0.6)
Asian	7,757 (44.4)	7,920 (46.6)	0.8 (0.7–0.9)
Hispanic	5,124 (29.3)	6,455 (38.0)	1.3 (1.2–1.5)
White	685 (3.9)	934 (5.5)	Referent
Other	481 (2.0)	338 (2.7)	0.7 (0.5–0.8)
**Age group (yrs)** ^¶^			
10–19	1,271 (7.3)	140 (0.8)	0.2 (0.2–0.3)
20–29	5,652 (32.3)	886 (5.2)	0.3 (0.3–0.3)
30–39	4,211 (24.1)	2,245 (13.2)	Referent
40–49	2,309 (13.2)	3,114 (18.3)	2.4 (2.2–2.6)
50–59	1,606 (9.2)	3,433 (20.2)	3.6 (3.3–3.9)
60–69	1,244 (7.1)	2,940 (17.3)	3.9 (3.6–4.3)
70–79	874 (5.0)	2,392 (14.1)	4.5 (4.0–4.9)
≥80	325 (1.9)	1,839 (10.8)	9.1 (8.0–10.5)
**Resident of correctional facility at time of diagnosis**	910 (5.2)	309 (1.8)	0.4 (0.4–0.5)
**Resident of long-term care facility at time of diagnosis**	91 (0.5)	297 (1.8)	1.6 (1.3–2.2)
Excess alcohol use within the previous year**	848 (4.9)	1,361 (8.1)	1.5 (1.3–1.6)
**Diabetes mellitus**	1,455 (8.3)	3,794 (22.3)	1.3 (1.2–1.4)
**HIV status at time of diagnosis**			
Positive	929 (5.3)	685 (4.0)	0.9 (0.8–1.1)
Unknown^††^	2,089 (11.9)	3,064 (18.0)	1.1 (1.0–1.2)
**Immunosuppression (not HIV/AIDS)^§§^**	325 (1.9)	880 (5.2)	1.6 (1.4–1.9)
**End-stage renal disease**	160 (0.9)	535 (3.2)	1.3 (1.1–1.6)
**TNF-α antagonist therapy**	47 (0.3)	131 (0.8)	2.2 (1.5–3.2)
**Previous organ transplantation**	18 (0.1)	121 (0.7)	2.5 (1.5–4.2)

**Abbreviations:** AIDS = acquired immunodeficiency syndrome; CI = confidence interval; HIV = human immunodeficiency virus; TNF-α = tumor necrosis factor alpha.

* Multivariable model: other characteristics investigated but not significant (p>0.05) in the univariate analysis included having extrapulmonary site of disease only, previous history of TB, being homeless within previous year, reporting injecting drug use within previous year, and reporting noninjecting drug use within previous year.

^†^ Odds ratios are for the association between each exposure variable and whether the patient had resided in the United States for ≥10 years or <10 years. Each odds ratio was adjusted for all of the other exposure variables displayed in the table using multivariable logistic regression.

^§^ Black, Asian, white and “other” are non-Hispanic. The “other” racial/ethnic category includes non-Hispanic Native Hawaiian and Other Pacific Islander, non-Hispanic American Indian/Alaskan Native, those of unknown race, and those reporting multiple races.

^¶^ Persons aged 0–9 years were excluded, because they could not have lived in the United States for ≥10 years.

** For variable definitions, refer to the following: CDC. CDC Tuberculosis Surveillance Data Training Report of Verified Case of Tuberculosis (RVCT) Self-Study Modules Participant Manual. Atlanta, GA: U.S. Department of Health and Human Services, CDC; 2009. https://www.cdc.gov/tb/programs/rvct/default.htm.

^††^ Laboratory HIV test was either refused or not offered or result was indeterminate or unknown or HIV status was unknown or missing.

^§§^ These data do not include HIV-infected patients, but patients who reported immunosuppression caused by either a medical condition or medication, or immunosuppressive therapy.

## Discussion

In recent years, more U.S. TB diagnoses among foreign-born persons occurred ≥10 years after arrival in the United States than among foreign-born persons in the United States <10 years. In 2013, for the first time, the number of TB cases diagnosed among foreign-born persons after ≥10 years in the United States was higher than the number diagnosed among persons in the United States for <10 years. Historically, TB prevention measures for foreign-born persons have focused on screening persons before or shortly after arrival in the United States and on finding and treating active TB disease (*6*). Although the joint effects of overseas and domestic TB prevention strategies are substantial, their independent effects on the trends of U.S. TB cases are unknown. Whereas TB case rates among foreign-born persons are highest among those who have newly arrived in the United States (*8*), rates of TB diagnosed among foreign-born persons ≥10 years after arrival remain substantially higher than those among U.S.-born persons. Most TB in the United States is thought to be a consequence of infection acquired years in the past, and recent estimates are that 92.5% of TB among foreign-born persons is caused by reactivation of LTBI (*1*). Therefore, most TB among foreign-born persons, even those who arrived ≥10 years ago, is probably attributable to infections acquired before U.S. arrival. These data support the recommendations by CDC and USPSTF to screen and treat persons with LTBI who were born in, or are former residents of, countries with increased TB prevalence regardless of time since arrival in the United States or age (*2*,*3*).

The findings in this report are subject to at least two limitations. First, NTSS does not routinely collect data regarding overseas travel by foreign-born patients since initial U.S. arrival; therefore, an unknown number of ≥10-year residents might have become infected with TB during more recent travel outside the United States. Second, data for month or year of first entry into the United States were missing for 9.1% of TB cases among foreign-born persons during 2010–2015. The majority of persons who reported year of U.S. entry without month information (and were therefore excluded from the comparison analysis) were among those in whom TB was diagnosed ≥10 years after U.S. arrival; if these persons had been included in this analysis, the number of TB cases diagnosed among foreign-born persons ≥10 years after U.S. arrival would have been even higher.

Historically, TB prevention activities in the United States have been implemented primarily by the public health sector (*9*). If CDC and USPSTF recommendations are implemented (*2*,*3*), prevention activities, including screening for TB infection through the use of the tuberculin skin test or interferon-gamma release assays, might need to be expanded beyond the public health sector to include private providers and community health centers to better reach populations that have lived in the United States for ≥10 years. The findings of this analysis that the diagnosis of TB in foreign-born persons ≥10 years after U.S. arrival is independently associated with being a resident of a long-term care facility and having non-HIV–related immunocompromising conditions (including, but not limited to, diabetes mellitus or end-stage renal disease) underscore the importance of LTBI screening and treatment to prevent TB disease in these populations. Continued initiatives for overseas and domestic screening as well as expanding partnerships with both private and public health care providers will be important in promoting testing and treatment for LTBI.

**Summary**

**What is already known about this topic?**

Tuberculosis (TB) screening in the United States of persons from high TB–prevalence countries has historically focused on newly arrived persons. U.S. TB cases typically occur among persons who were infected years before experiencing disease. Persons with latent TB infection have a 5%–10% lifetime risk for developing TB disease in the United States.

**What is added by this report?**

Beginning in 2013, the number of TB diagnoses among foreign-born persons ≥10 years after U.S. arrival (2,823) has exceeded those among persons <10 years after U.S. arrival (2,814). In 2015, among 5,763 TB cases diagnosed in foreign-born persons in the United States for whom the date of U.S. entry was known, 2,922 (51%) were diagnosed in persons ≥10 years after U.S. arrival. Foreign-born persons who received a TB diagnosis ≥10 years after U.S. arrival had greater odds of being aged ≥40 years, residing in a long-term care facility at diagnosis, and having non-HIV–related immunocompromising conditions.

**What are the implications for public health practice?**

Promoting testing for TB infection as part of routine primary care among groups at high risk is crucial for advancing TB prevention and elimination initiatives in the United States. Emphasis should be focused on persons who have lived in countries with high TB prevalence, including persons who have resided in the United States for ≥10 years.

## Footnotes

* The U.S. insular areas are American Samoa, Guam, Puerto Rico, U.S. Virgin Islands, and Commonwealth of the Northern Mariana Islands.

† The freely associated states are the sovereign nations that have signed compacts of free association with the United States (Federated States of Micronesia, Republic of the Marshall Islands, and Republic of Palau).
